# Forecasting Battery Electrode Performance via Electrochemical Fluorescence Microscopy and Machine-Learning

**DOI:** 10.1021/acsami.5c17708

**Published:** 2025-12-03

**Authors:** Karla Negrete, Marco-Tulio Fonseca Rodrigues, Daniel P. Abraham, Maureen H. Tang

**Affiliations:** † Department of Mechanical Engineering & Mechanics, 6527 Drexel University, Philadelphia, Pennsylvania 19104, United States; ‡ Chemical Sciences & Engineering Division, 1291 Argonne National Laboratory, Lemont, Illinois 60439-4801, United States; § Department of Chemical & Biological Engineering, Drexel University, Philadelphia, Pennsylvania 19104, United States

**Keywords:** electrochemical fluorescence microscopy, machine learning, battery electrodes, performance predictions, data-driven manufacturing

## Abstract

Predicting lithium-ion battery performance is hindered by microscale electrode heterogeneities invisible to conventional diagnostics. Here, we combine electrochemical fluorescence microscopy (EFM), which maps electronic connectivity by visualizing an electrofluorophore reaction distribution, with a multitask ElasticNet regression to forecast discharge capacity from spatial heterogeneity. Analyzing 196 images from six pilot-scale LiNi_0.5_Mn_0.3_Co_0.2_O_2_ cathodes with varying carbon loadings, we extract 62 descriptors that capture morphology and texture. A compact five-feature model predicts capacity across eight discharge rates, achieving a per-target *R* ^2^ of up to 0.63 and an overall *R* ^2^ of 0.92, with a mean absolute percentage error of less than 2%. This performance rivals impedance-based approaches while avoiding their reliance on postformation data and incomplete electronic network information. Our facile and rapid, image-driven method may enable electrode quality control upstream of costly cell assembly to offer a transformative tool for data-driven battery research and manufacturing.

## Graphical abstract

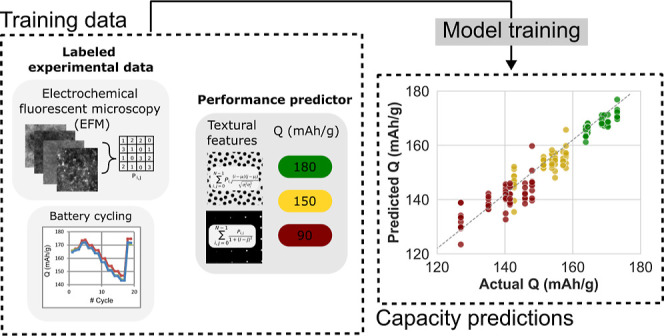

## 1 Introduction

Despite tremendous importance to electric vehicles and consumer devices, predicting the performance of lithium-ion batteries (LIBs) remains challenging due to microscale heterogeneities in electrode structure that evade conventional diagnostics.^1,2^ Nominally identical cells can diverge widely in capacity and lifespan, with the weakest units limiting module safety and reliability.^3^ Variations in the spatial distribution of active material, conductive carbon, and binder within the cathode influence electronic connectivity but remain invisible to bulk electrochemical measurements.^4–6^ While machine learning (ML) has shown promise for state estimation and degradation forecasting,^7–15^ most approaches rely on time-series voltage or current data that are path-dependent, low-resolution, and unavailable until after significant cycling.^16,17^ This dependence on time-series data limits their utility for early stage quality control. A predictive framework that can link electrode-level heterogeneity to downstream performance, before cell assembly and formation, which account for over 60% of manufacturing cost,^18^ could transform battery production by enabling proactive image screening of suboptimal electrodes before they enter costly electrochemical workflows.

To address this gap, we introduce an image-based framework that couples electrochemical fluorescence microscopy (EFM) with a penalized multitask regression model to forecast discharge capacity directly from mesoscale spatial heterogeneity. In EFM, an electrofluorophore emits light only where continuous electronic networks exist, revealing connectivity with high spatial resolution. We applied this approach to six pilot-scale LiNi_0.5_Mn_0.3_Co_0.2_O_2_ cathodes with carbon black loadings from 1–5 wt %, yielding 196 fluorescence images. From each image, we extracted 62 statistical descriptors capturing spatial autocorrelation, texture, and morphology. Trained solely on these descriptors, our model achieves per-target *R* ^2^ values up to 0.63 and an overall *R* ^2^ of 0.92 with mean absolute percent error below 2%, rivaling more complex, multimodal methods that depend on post-formation electrochemical or impedance data.^19–22^ The consistent selection of a compact, five-feature set across all targets underscores the model’s stability and interpretability. By relying solely on intensity-based texture and blob-derived morphological features, our framework enables early stage, facile, and rapid quality control, paving the way for predictive diagnostics upstream of formation and accelerating the path toward data-driven manufacturing.

Figure 1 illustrates the operating principle of EFM. The cross-section of a composite cathode depicts suboptimal manufacturing, in which some particles of active material form strong electronic connections with conductive carbon and binder (left) while others are electronically isolated (right). Electronically isolated regions of electrodes are not only dead weight/volume within the battery, which negatively effects overall performance metrics, but also regions which can experience extreme potentials during cycling, leading to side reactions that can generate gases and other detrimental products. In EFM, the optical cell is filled with an electrolyte containing a reversible electrofluorophore that fluoresces only upon reduction at 1.95 V vs Li/Li^+^, well below typical cathode lithiation (∼3.0 V vs Li/Li^+^). Polarization at −7.6 mA/cm^2^ selectively reduces the fluorophore in electronically connected regions, allowing spatial mapping of electronic accessibility.

**Figure 1:**
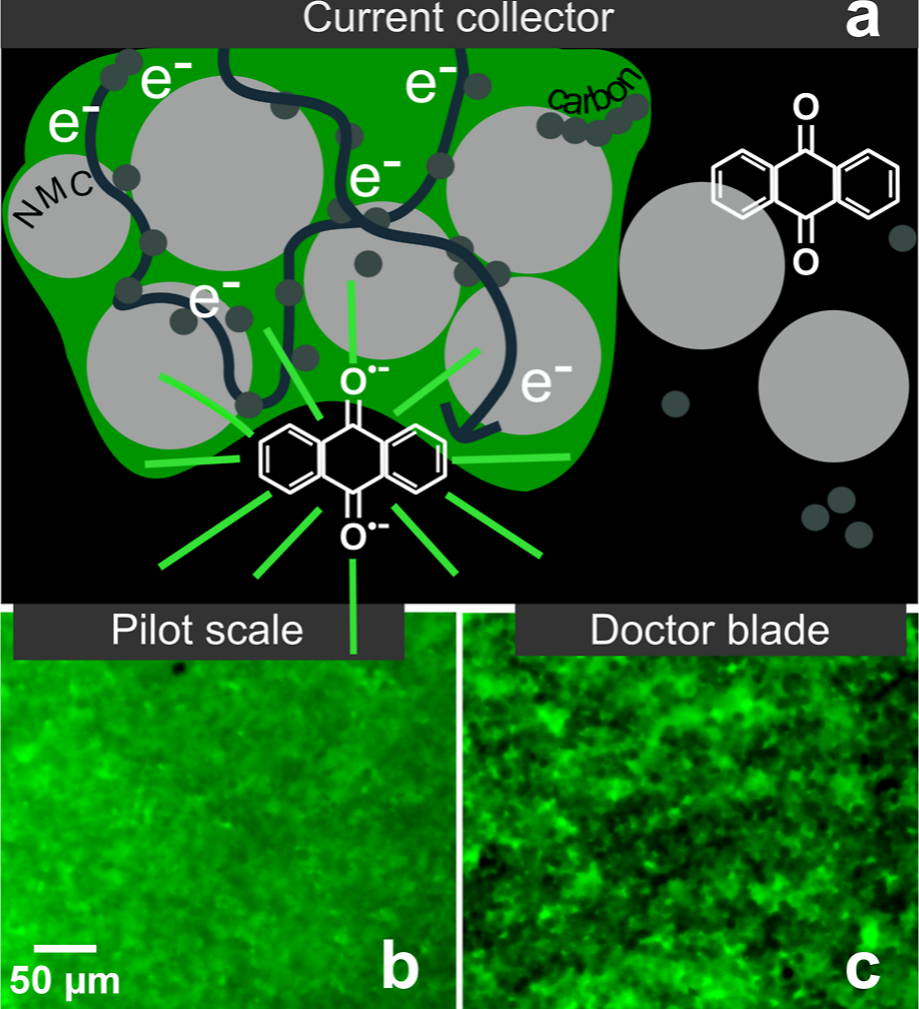
The EFM mechanism.^23^ Cross-sectional schematic of an electrode (a): homogeneous regions within the composite transfer electrons to the electrofluorophore in the electrolyte, producing fluorescence. Bright areas in top-down images indicate strong electronic connectivity; dark regions reveal electronically isolated or disconnected particles. EFM images comparing the top surface of two NMC electrodes: (b) high-performance electrode from CAMP pilot-scale facility and (c) low-performance electrode fabricated in-house via doctor blade.

Under the microscope, fluorescence intensity directly reports the concentration of reduced fluorophore, which corresponds to the accessibility of the electronic network. Bottom panels of Figure 1 contrast EFM images of high- and low-quality NMC electrodes. An electrode made with a pilot-scale coater at Argonne’s cell analysis modeling and prototyping (CAMP) facility exhibits near-uniform fluorescence with subtle gradients. In contrast, a doctor-bladed, lab-scale electrode shows pronounced dark regions, previously linked to electronically isolated particles.^23–25^ Because EFM probes only the top surface, where electronic connectivity is likely lowest, observed heterogeneity therefore provides a conservative metric of network quality.

While qualitative variations in Figure 1b,c reflect differences in manufacturing scale and process control, our goal was to move beyond visual inspection and quantitatively compare heterogeneity among high-quality, pilot-scale electrodes. Although our prior work correlated EFM fluorescence with capacity fade in lab-scale composites, its application to industrially relevant electrodes and its potential to yield predictive heterogeneity descriptors remains untested. Here, we extract 62 image-derived heterogeneity features from the CAMP library of pilot-scale electrodes and pair them with half-cell rate-performance data. We show that heterogeneity metrics alone can predict discharge capacity, establishing a new approach to performance evaluation in electrode manufacturing.

## 2 Results and Discussion

Six pilot-scale electrodes were fabricated at Argonne’s CAMP facility using BASF TODA NMC532 active material and SOLVAY 5130 PVDF binder, varying only in carbon black type: Timcal Super C45 (t) or Cabot LITX 200 (c), at loadings from 1 to 5 wt % (Figure 2). Formulation labels such as “90:5­(t):5” denote 90 wt % NMC532, 5 wt % carbon, and 5 wt % PVDF. The medium-structure Super C45 network typically requires 2 to 5 wt % to percolate reliably. In contrast, the high-structure LITX 200 achieves percolation at 1 to 3 wt %, potentially enabling higher active-material content and improved energy density.^26,27^ These differences are evident when comparing equivalent carbon content (96:2:2): cells with LITX 200 consistently outperform Super C45 in discharge capacity (Supporting Information Figure S1). EFM images (lower panels, Figure 2a) reveal uniform fluorescence at 5 wt %, with increasing fragmentation (“peppered” dark regions) at 2 to 4 wt %, evolving into discrete disconnected islands below 1.5 wt %.

**Figure 2:**
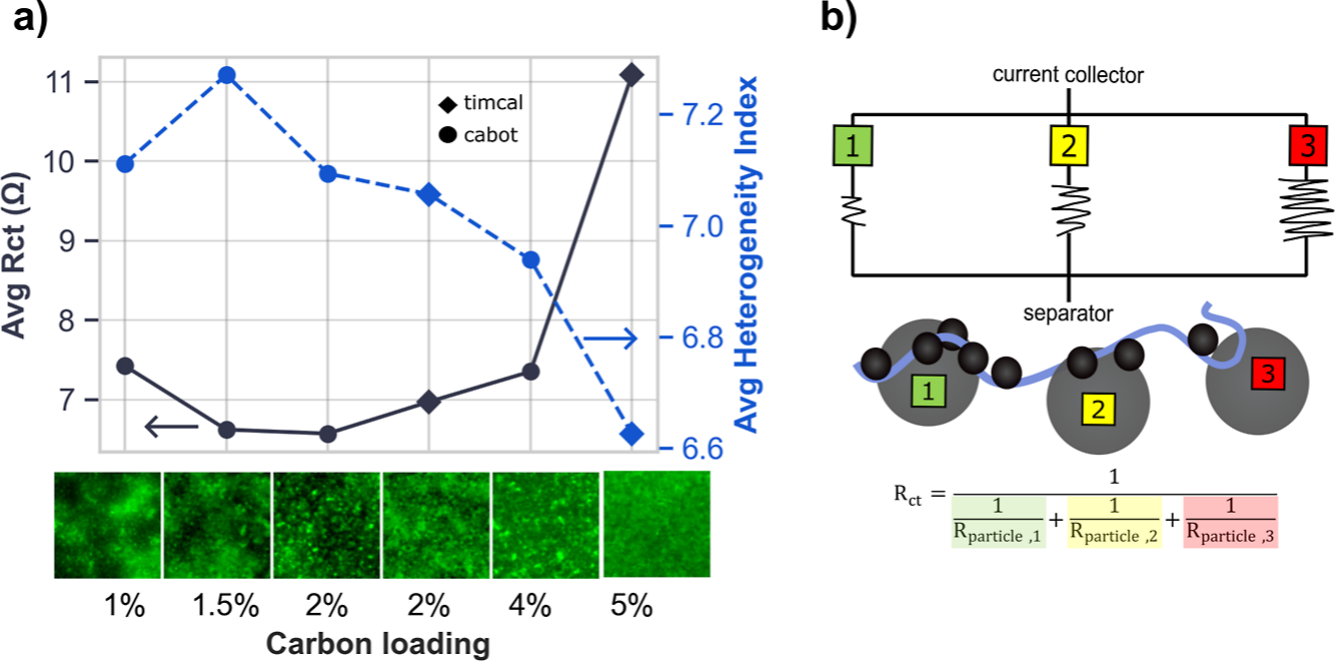
Contrasting views of electronic transport in composite electrodes. (a) Six NMC532 cathodes were fabricated with Timcal Super C45 (diamond) or Cabot LITX 200 (circle) at 1–5 wt % carbon. EFM images quantify a fluorescence-derived heterogeneity index that decreases with carbon loading, whereas charge-transfer resistance (*R*_ct_) from EIS paradoxically increases. (b) Schematic illustration of parallel resistors shows why EIS underestimates heterogeneity: only particles well connected to the carbon binder domain contribute to the measured *R*_ct_, while disconnected regions remain invisible. EFM, by contrast, resolves both connected and disconnected domains, directly capturing transport inhomogeneities.

We quantified heterogeneity using Shannon entropy, here termed the heterogeneity index, computed from the normalized 256-bin intensity histogram of each image. Entropy values spanned 6.6 bits (most uniform, 5 wt %) to 7.3 bits (most heterogeneous, 1.5 wt %), where increments of 0.1 bit correspond to perceptible shifts from continuous fluorescence to isolated dead zones (Figure 2a). By contrast, charge-transfer resistance (*R*_ct_) measured by EIS exhibits the opposite trend, in which the 5 wt % formulation shows nearly double the *R*_ct_ of lower-loading electrodes. This discrepancy occurs because EIS is a bulk, low-perturbation probe that lacks spatial resolution. It preferentially reports the lowest-resistance pathways, akin to the brightest hotspots in EFM, while overlooking inactive regions. Consequently, it systematically underestimates the true extent of transport limitations. As Newman has shown, standard interpretations of *R*_ct_ in porous composites are misleading without explicit percolation models.^4^ Figure 2b illustrates this concept: active particles act as resistors in parallel, but only those well connected to the carbon binder domain contribute to the measured *R*_ct_. This measurement inflates the apparent connectivity of the film and conceals resistive regions of the underlying electronic network. By contrast, the fluorescence-derived heterogeneity index from EFM captures both connected and disconnected domains and directly reveals electronic transport inhomogeneities that EIS cannot access.

From 196 high-magnification EFM images, we extracted 62 quantitative heterogeneity descriptors spanning global spatial statistics, patch-wise variation, radiomic texture, and blob metrics, indexed by electrode formulation. To remove redundancy, we computed Pearson correlation coefficients across descriptors and discarded one member of each pair with |*r*| > 0.85, yielding 15 independent descriptors (Supporting Information Figure S4). For formulation-resolved analysis, each feature was *Z*-score normalized across formulations to enable direct comparison of heterogeneous metrics. Figure 3 highlights the most diagnostic descriptors across carbon loadings: blob count (connected-component analysis of dark particle-like regions), gray-level run length matrix (GLRLM, low gray-level run emphasis), gray-level co-occurrence matrix (GLCM, sum average), heterogeneity index (Shannon entropy), Moran’s I (global spatial autocorrelation), and local binary pattern with radius = 1 and 8 neighbors (LBP_*R*=1,*P*=8_, energy statistic). We further examine the individual and cumulative contributions of these top features to model predictions using SHAP (SHapley Additive exPlanations) analysis, where features with higher absolute mean SHAP values are deemed more important. As shown in Supporting Information Figure S5, blob count is the most influential single feature, with an absolute mean SHAP value of 0.4, whereas the combined effect of the top six features reaches 0.9.

**Figure 3:**
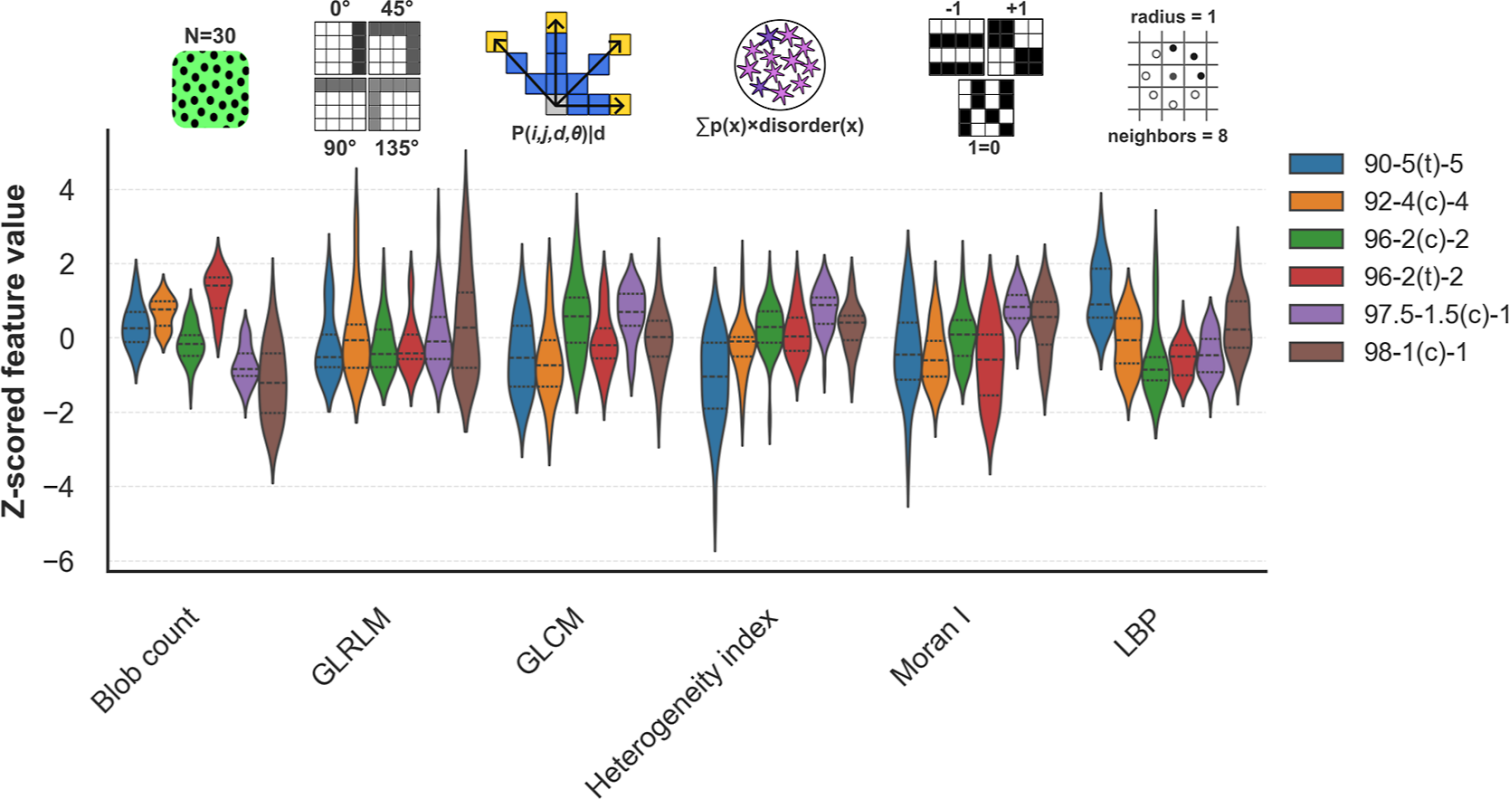
Feature variability across electrode formulations. Violin plots display the distribution of selected image-derived features after *z*-scoring, highlighting formulation-dependent trends. Features include blob count (disconnected active material particle density), GLRLM (gray-level run-length measures of texture), GLCM (co-occurrence-based texture metrics), heterogeneity index (Shannon entropy of the intensity histogram), Moran’s I (spatial autocorrelation), and LBP (local binary patterns, pixel neighborhood descriptors). Each color corresponds to a distinct electrode formulation, with carbon loading and source specified in the legend. Shifts in median position capture systematic differences between formulations. For example, blob count, heterogeneity index, and Moran’s I vary strongly with carbon loading, whereas other descriptors such as GLRLM reveal more subtle but consistent formulation-dependent trends.

The top three descriptors are blob count, the heterogeneity index, and Moran’s I. Blob count quantifies discrete low-intensity regions after thresholding; higher blob counts at low carbon loadings are consistent with a weaker electronic network and fewer actively connected particles. The heterogeneity index and Moran’s I are global descriptors that capture complementary aspects of large-scale intensity structure. A higher heterogeneity index denotes a broader, more disordered intensity distribution, indicating increased electronic heterogeneity. Moran’s I measures global spatial autocorrelation: values near +1 indicate spatially continuous domains, near 0 randomness, and near −1 checkerboard-like alternation. In our images, elevated Moran’s I at low carbon loadings reflects clustered, fluorescence-rich “islands” of connected active particles separated by dark regions of isolated active particles. Texture descriptors provide complementary, scale-dependent information. LBP_*R*=1, *P*=8_ energy measures the concentration of local binary patterns: high energy indicates dominance of a few repeating microtextures, whereas low energy indicates diverse local motifs. Experimentally, higher LBP energy at increased carbon content corresponds to the fine “peppering” of intensity that we establish as the baseline homogeneity. GLRLM quantifies consecutive runs of identical gray levels along multiple directions (0°, 45°, 90°, 135°); the predominant GLRLM metric here is low gray-level run emphasis (LGRE), which up-weights runs at darker intensities. Because LGRE pools contributions from both short and long low-intensity runs, its median may be less sharply separated across loadings even though it explains substantial image variability. Finally, the GLCM SumAverage (averaged over 0°, 45°, 90°, 135°) captures the expected summed intensity of neighboring pixel pairs: differences in SumAverage arise from the bright-connected islands and dark isolated regions at low carbon versus the more uniformly peppered intensity field at higher carbon. Defining such formulation-resolved trends in image-derived heterogeneity features offers substantial value for quality control in electrode fabrication. Given that fabrication accounts for ∼45% of cell-production cost^28^ and that a 5.3 GWh yr^–1^ plant incurs $140–180 million USD GWh^–1^ annually,^29^ even minor improvements in quality control can yield substantial savings.

Although formulation-resolved trends are informative, our primary aim was to establish a proof-of-concept: can a compact set of image-derived heterogeneity features predict discharge capacity under stringent modeling constraints? From 196 EFM images, each paired with the mean capacity of its electrode formulation (six formulations total), we regressed 15 image features against capacities at eight protocol stages (C/25 to 2C). With only six distinct formulations, the problem is underdetermined for ordinary least-squares (OLS), since the number of predictors exceeds the number of independent inputs. To address this, we implemented a penalized multitask ElasticNet, which jointly learns across all eight discharge rates and enforces shared sparsity to stabilize estimates. Nested, stratified 5-fold cross-validation with a held-out test partition was used, with all preprocessing, feature selection, and hyperparameter tuning confined to the inner folds (Supporting Information Figure S6). The procedure selected α = 0.0336 and l1_ratio = 0.9, where the l _1_ term eliminates noninformative features by driving coefficients to zero. The l _2_ term shrinks the remaining coefficients toward each other to stabilize estimates and preserve correlated predictors. With l1_ratio = 0.9, the model strongly favors sparsity while retaining some grouping.

Despite the demanding regime, the multitask ElasticNet model consistently selected only 3–5 features (well below the number of formulations) and outperformed single-task OLS in cross-validation by Δ*R* ^2^ ≈ 0.10, while reducing fold-to-fold variance 4-fold (Supporting Information Figure S7). On the independent hold-out set, per-target *R* ^2^ reached up to 0.63 (C/25) and 0.61 (second C/25 stage) with relative error <1.2% and RMSE < 2.6. Across faster rates the model achieved *R* ^2^ = 0.51 to −0.60 (C/10 to 2.0C) (Figure 4). Aggregated across all eight targets, the final model yields *R* ^2^ = 0.92, RMSE = 3.53, and mean absolute error <2%. Supplementary Table S3 reports the fully fitted equations along with the corresponding feature weights. This level of performance is consistent with or surpasses impedance-based approaches. Zhang et al. achieved *R* ^2^ ≈ 0.90 with mean absolute percent error ∼2.0% using full EIS spectra.^19^ Liu et al. reported lower RMSE (∼1.1%) using variational autoencoders on impedance data.^22^ In contrast, Jones et al. demonstrated probabilistic capacity forecasts with higher test error (∼8.2%) from single-scan EIS.^20^ Our image-based regression achieves comparable or superior accuracy while avoiding the limitations of EIS, which probes only part of the electrode’s electronic network.

**Figure 4:**
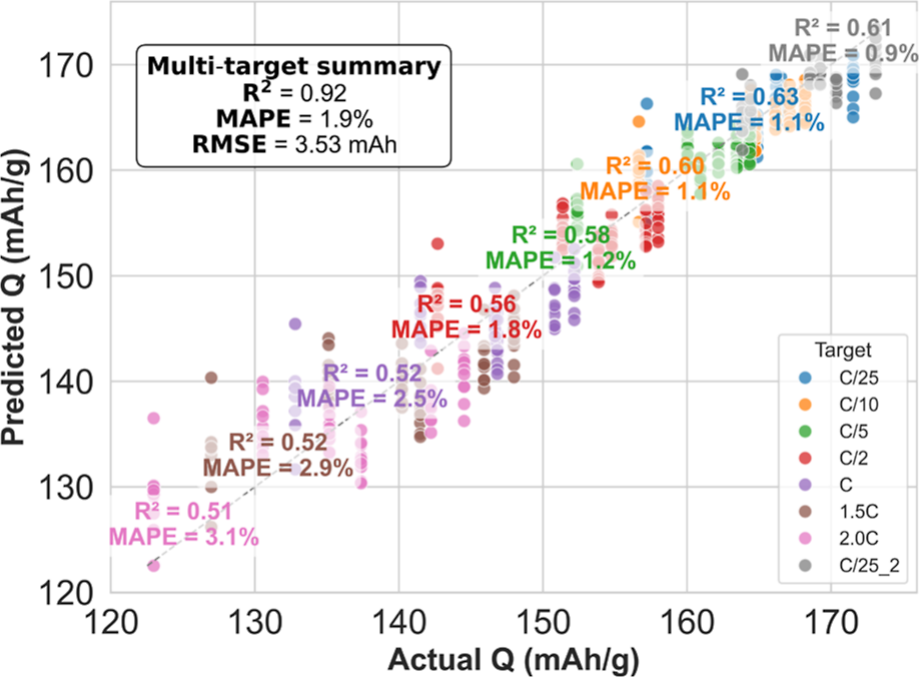
Predicted vs measured discharge capacities across rate protocols. Each point shows a formulation-level prediction from the multitask ElasticNet model, trained on 15 image features. Despite only six unique formulations, the model selects 3 to 5 predictors and achieves test *R* ^2^ up to 0.63, with errors under 3% and RMSE from 2.06 to 4.97 mAh. Overall, the model attains an aggregate *R* ^2^ = 0.92, RMSE of 3.53 mAh, and mean absolute error below 2% across all rate conditions.

To address concerns of information leakage due to shared formulation-level capacities, we also attempt a leave-one-formulation-out analysis. In this approach, all images from one formulation are held out for testing while training is conducted on the remaining formulations, ensuring strict independence between training and test data. While conceptually the most conservative approach, leave-one-formulation-out is less suited to this compact six-formulation data set, as it reduces variability within the training folds. Comparing the workflow (Supporting Information Figure S8) and results (Supporting Information Figure S10) confirm that, as expected, the leave-one-formulation-out framework can predict performance when interpolating for intermediate carbon loadings (1.5%, 2%, and 4%), but accuracy degrades when extrapolating to carbon loadings (5% and 1%).

## 3 Conclusion

In summary, this work has demonstrated that EFM can predict electrode performance from *ex situ* visualizations of local electronic connectivity. EFM requires relatively inexpensive and accessible equipment and can be obtained before cell assembly or cycling. Here, we show that under stringent formulation constraints, a small set of image-derived features can predict discharge capacity with accuracy comparable to state-of-the-art electrochemical methods. Our proof-of-concept offers promising evidence that expanding this framework to broader formulation diversity and true cell-to-cell variability could generalize relationships across materials, architectures, and manufacturing conditions. Performance predictions via EFM may expedite battery R&D by reducing the number of cell experiments required to determine the viability of electrode materials or formulations. For manufacturing quantity control, EFM may offer robust classification to detect defected electrodes upstream of cell assembly for faster feedback and reduced scrap rates. Scaling and validating this approach will require collaboration among national laboratories, academia, and industry, establishing the foundation for performance-guided optimization in electrode manufacturing. Ultimately, a closed-loop data-driven manufacturing design will rely on frameworks such as this one to connect electrode fabrication, electronic structure, and battery performance.

## 4 Experimental Section

### 4.1 Electrode Preparation and Battery Cycling

#### 4.1.1 Electrode Formulations

Six pilot-scale cathode formulations were fabricated at Argonne’s CAMP Facility using BASF TODA NMC532 active material and Solvay 5130 PVDF binder. The formulations varied only in carbon black type, either Timcal Super C45 (BET ≈ 45 m^2^/g, OAN ≈ 36 mL/100 g) or Cabot LITX 200 (BET 150–1500 m^2^/g, OAN 100–200 mL/100 g), and in loading, ranging from 1 to 5 wt %. Slurries were prepared in *N*-methylpyrrolidone (NMP), cast onto 20 μ m aluminum foil, dried, and calendered to a final thickness of 53–71 μ m, corresponding to 34.8–35.6% porosity. Final blends spanned a formulation range from an additive-heavy 90:5:5 to a lean 98:1:1 (active/carbon/binder, wt %) blend.

#### 4.1.2 Coin Cell Assembly

Cathodes were punched into 14 mm disks and dried under dynamic vacuum at 120 °C overnight. 2032-format coin cells were assembled using Li metal anodes (15 mm), Celgard 2500 separators (16 mm), and 40 μL of the electrolyte (1.2 M LiPF_6_ in EC/EMC, 3:7 wt %). All components were handled in a dry room to minimize moisture uptake.

#### 4.1.3 Electrochemical Measurements

Cathode capacities were evaluated on a Maccor Series 4100 cycler between 2.5 and 4.3 V. For each formulation (10–16 cells), rate capability was assessed with two cycles each at C/25, C/10, C/5, C/2, 1, 1.5, and 2C, followed by two final C/25 cycles to re-establish baseline. Each charge half-cycle terminated with a hold at 4.3 V until the current had decayed to below C/25. Discharge was always performed under constant current. A 2 min rest followed each half-cycle. Capacities are reported per gram of active material.

Electrochemical impedance spectroscopy (EIS) was performed on triplicate samples for each cathode. These tests used full-cells, obtained by pairing each cathode with the same type of CAMP-fabricated anode (91.8 wt % Superior Graphite SLC1506T, 2 wt % Timcal C45, 6 wt % PVDF, 0.17 wt % oxalic acid). Spectra were collected after five cycles (3x C/10, 2x C/2), and after equilibration of the cells at 3.8 V.

### 4.2 Electrochemical Fluorescent Microscopy

#### 4.2.1 Optical Cell Assembly

Electrochemical fluorescent microscopy (EFM) experiments were conducted in a modified ECC-Opto-10 optical cell (El-Cell) fitted with a 1.1 mm-thick FTO conductive window (Saida Glass Co.). The cell was assembled face-to-face, with the cathode at the bottom. A 25 μ m PTFE separator (McMaster-Carr), laser-cut with nine 1 mm openings, was placed above the cathode. The cell was flooded with an electro-fluorophore electrolyte (1 mM 9,10-anthraquinone in 0.1 M TEABF_4_/propylene carbonate; Millipore Sigma) and sealed in an Ar-filled glovebox (LC Tech).

#### 4.2.2 In Situ Imaging

Fluorescence imaging was performed on a ZEISS Axio Observer wide-field microscope coupled to a BioLogic potentiostat. A custom filter cube (375 nm excitation, 515 nm emission) and an LD Plan-Neofluar 20×/0.4 objective were used. A cathodic current of 60 μA activated the fluorophore, and nine regions of interest (ROI, 1 mm apertures) were imaged with 20 s exposures to minimize photobleaching. For each formulation, three to five electrodes were imaged, resulting in a maximum of 45 images per formulation. The ROIs were selected across the electrode surface to capture representative spatial variation and minimize sampling bias, ensuring that the quantitative fluorescence measurements reflected the overall heterogeneity of the electrode.

#### 4.2.3 ML Framework

The workflow included five sequential steps: (1) feature extraction, (2) data set generation, (3) feature reduction, (4) model training and prediction, and (5) model evaluation. All computations were implemented in Python 3.10 using standard libraries.^30–32^

##### 4.2.3.1 Feature Extraction

Feature extraction was timed to identify bottlenecks and ensure scalability. Timing data and extracted features were organized by sample and archived for transparency and reproducibility. Features incorporated into the ML models are highlighted in bold in the descriptions that follow. A comprehensive overview of the extracted features with corresponding references and timing results is provided in Supporting Information Tables S1 and S2.

#### 4.2.4 Preprocessing

Grayscale TIFF images were normalized to the range [0, 1], resized to 429 × 563 px via bicubic interpolation, and downconverted to 8 bit depth for uniform spatial resolution. A full-frame binary mask defined the analysis region. Features were standardized to have a mean of zero and a variance of one before modeling.

#### 4.2.5 Global Spatial Statistics

Four whole-image descriptors quantified spatial heterogeneity.

Moran’s I measured spatial autocorrelation across 8-connected neighborhoods

$$I=\frac{N}{\sum_{i,j}w_{ij}}\cdot \frac{\sum_{i,j}w_{ij}\left(x_{i}-\mathrm{x̅}\right)\left(x_{j}-\mathrm{x̅}\right)}{\sum_{i}{\left(x_{i}-\mathrm{x̅}\right)}^{2}}$$

where *N* is the number of pixels, *x*_*i*_ the intensity at pixel *i*, x̅ the mean image intensity, and *w*_*ij*_ the spatial weight (1 if pixels *i*, *j* are neighbors, 0 otherwise). Values near +1 indicate strong clustering, while values near −1 indicate dispersion.^33^

Shannon entropy quantified grayscale disorder using a 256-bin histogram

$$H=-\sum_{k=1}^{256}p_{k}\log_{2}p_{k}$$

where *p*_*k*_ is the normalized probability of intensity level *k*. Higher *H* reflects greater grayscale heterogeneity.^34^

Global mean intensity μ and standard deviation σ summarized overall brightness and variability

$$\mu =\frac{1}{N}\sum_{i}x_{i},,\sigma =\sqrt{\frac{1}{N}\sum_{i}{\left(x_{i}-\mu \right)}^{2}}$$

#### 4.2.6 Patch-wise Variability

To probe mesoscale heterogeneity, images were divided into nonoverlapping 70 × 70 px tiles (22 × 22 μm). For each valid tile, we computed

$$\mu_{t}=\frac{1}{n_{t}}\sum_{i\in t}x_{i},,\sigma_{t}^{2}=\frac{1}{n_{t}}\sum_{i\in t}{\left(x_{i}-\mu_{t}\right)}^{2},,H_{t}=-\sum_{k=1}^{256}p_{k,t}\log_{2}p_{k,t}$$

where *n*_*t*_ is the number of pixels in tile *t*, and *p*_*k*,*t*_ is the normalized histogram of intensities. Global patch descriptors are averages across all tiles: $\mu_{patch}=\frac{1}{T}\sum_{t}\mu_{t}$ , $\sigma_{patch}^{2}=\frac{1}{T}\sum_{t}\sigma_{t}^{2}$ , $H_{patch}=\frac{1}{T}\sum_{t}H_{t}$ .

#### 4.2.7 Radiomic Texture Descriptors

Second-order and frequency-domain descriptors were extracted using PyFeats:^31^

- GLCM (Gray-Level Co-occurrence Matrix): For a given offset (Δ*x*, Δ*y*), the co-occurrence matrix entry is

P(i,j)=#{(x,y)|I(x,y)=i,I(x+Δx,y+Δy)=j}

where *I*(*x*, *y*) is the gray level at pixel (*x*, *y*). The matrix is normalized such that

$$\sum_{i=1}^{G}\;\sum_{j=1}^{G}P\left(i,j\right)=1$$

where *G* is the number of gray levels.

Haralick features^35^ are derived from *P*(*i*, *j*). For example, the *sum average* is

$$f_{sum\text{\_}avg}=\sum_{k=2}^{2G}kp_{x+y}\left(k\right),,p_{x+y}\left(k\right)=\sum_{i+j=k}P\left(i,j\right)$$

Features were computed for four directions (0°, 45°, 90°, 135°) and averaged.

- GLRLM (Gray-Level Run Length Matrix): The run-length matrix *R*(*i*, *j*) records the number of contiguous runs of gray level *i* with run length *j* along a given direction. Formally,

R(i,j)=#{runsofgrayleveliwithlengthj}

here *i* ∈ {1, ..., *G*} indexes gray levels and *j* ∈ {1, ..., *R*} indexes run lengths, where *G* is the number of gray levels and *R* the maximum run length. The total number of runs is

$$N_{r}=\sum_{i=1}^{G}\;\sum_{j=1}^{R}R\left(i,j\right)$$

From *R*(*i*, *j*), several statistics are defined.^36,37^ The low gray-level run emphasis (LGRE) emphasizes contributions from low-intensity pixels

$$LGRE=\frac{1}{N_{r}}\sum_{i=1}^{G}\;\sum_{j=1}^{R}\frac{R\left(i,j\right)}{i^{2}}$$

LGRE attains larger values when runs of darker pixels dominate, irrespective of run length, thus capturing the prevalence of low-intensity regions.

- LBP (Local Binary Patterns): For radius *R* and *P* neighbors,

$${LBP}_{R,P}\left(x_{c}\right)=\sum_{p=0}^{P-1}s\left(I\left(x_{p}\right)-I\left(x_{c}\right)\right)2^{p},,s\left(z\right)=\{\begin{array}{ll} 1, & z\geq 0\\ 0, & z<0 \end{array}$$

where *x*_*c*_ is the center pixel. Histogram-based statistics, such as energy, were computed.

- NGTDM, GLDS, SFM: Capturing neighborhood differences, gray-level differences, and submatrix statistics, respectively, each defined per.^31^
- LTE (Laws’ Texture Energy): Convolution with 7-tap Laws’ kernels (e.g., L5, E5, S5) yields energy maps; the mean energy per filter characterizes primitive textures (edges, spots, waves).
- Fourier Power Spectrum (FPS): The 2D Fourier transform *F*(*u*, *v*) yields radial energy

$$P\left(r\right)=\frac{1}{\left|\Omega \left(r\right)\right|}\sum_{\left(u,v\right)\in \Omega \left(r\right)}{\left|F\left(u,v\right)\right|}^{2}$$

averaged across annuli Ω­(*r*), capturing periodicity and frequency content.

##### 4.2.7.1 Data Set Generation

Extracted feature vectors were compiled into a data set indexed by cathode formulation, with discharge capacities from eight protocol steps serving as multivariate targets. Given the relatively small sample size and diversity across electrode types, a stratified split was used to partition the data into training (70%) and validation (30%) subsets while preserving the distribution of cathode formulations. This ensured that performance metrics reflected generalization across all sample types, rather than overfitting to dominant classes.

##### 4.2.7.2 Feature Reduction

To mitigate multicollinearity, features with a Pearson correlation coefficient exceeding |*r*| > 0.85 were removed based on the upper triangle of the correlation matrix computed from the training set. This reduced the original 62 features to a set of 15 statistically independent predictors. No additional feature selection or engineering was required, as the sparsity-inducing regularization of the learning algorithm automatically pruned redundant or noninformative inputs during training.

##### 4.2.7.3 Model Training and Prediction

Although the data set contained 196 image samples, these represented only six unique cathode formulations, limiting the effective number of independent inputs. After feature reduction, 15 predictors remained, exceeding the number of unique inputs, and preventing standard linear regression methods such as ordinary least-squares (OLS) from reliably finding stable solutions. While ElasticNet regularization reduces the number of active features, fitting separate models for each electrochemical protocol step can lead to overfitting.

To address this, we adopted a multitask learning framework in which discharge capacities from all protocol steps were modeled jointly. By learning multiple related outputs simultaneously using a shared set of predictors, the model captures commonalities across tasks, thereby improving stability and generalization.^38^ All features and targets were standardized to zero mean and unit variance; target scaling was reversed after prediction to report performance in the original units.

The multitask elastic net estimates the coefficient matrix $W\in R^{p\times T}$ by solving

$$\arg\min_{W}\sum_{i=1}^{n}\sum_{t=1}^{T}{\left(y_{i,t}-{ŷ}_{i,t}\right)}^{2}+\lambda \left[\alpha {∥W∥}_{2,1}+\left(1-\alpha \right){∥W∥}_{F}^{2}\right]$$

where *y*_*i*,*t*_ and ${ŷ}_{i,t}$ are the observed and predicted values for sample *i* and task *t*. The term ∥**W**∥_2,1_ is a group-lasso norm promoting shared sparsity across tasks, while ∥**W**∥_*F*_^2^ is the squared Frobenius norm providing ridge regularization. The parameters λ and α control overall regularization strength and the balance between sparsity and shrinkage, respectively.

Hyperparameters λ and α were optimized via grid search using stratified cross-validation to preserve the distribution of cathode formulations across folds. The final model was trained on the whole training set using the best-performing parameters and evaluated on a held-out test set to assess generalization.

##### 4.2.7.4 Model Evaluation

Model performance was assessed using nested 5-fold stratified cross-validation, where inner folds were used for hyperparameter tuning and outer folds evaluated generalization on held-out data. For each output, we report the coefficient of determination

$$R^{2}=1-\frac{\sum_{i=1}^{N}{\left(y_{i}-{ŷ}_{i}\right)}^{2}}{\sum_{i=1}^{N}{\left(y_{i}-\mathrm{y̅}\right)}^{2}}$$

the root-mean-square error (RMSE)

$$RMSE=\sqrt{\frac{1}{N}\sum_{i=1}^{N}{\left(y_{i}-{ŷ}_{i}\right)}^{2}}$$

and the mean absolute percent error (MAPE)

$$MAPE=\frac{100}{N}\sum_{i=1}^{N}\left|\frac{y_{i}-{ŷ}_{i}}{y_{i}}\right|$$

These metrics were computed separately per output and then averaged across outer folds.

To provide a summary of overall model accuracy across all targets, predictions, and actual values were concatenated across outputs and evaluated jointly. This approach produces global *R* ^2^, RMSE, and MAPE scores that reflect performance across the entire output space, accounting for the relative variance and scale of each target. Per-target and overall results are presented in Supporting Information Table S3.

## Supplementary Material



## Data Availability

196 TIFF images, parameter metrics, and Python scripts are openly available under an open-source license at: https://github.com/karnegre/efm-ml-framework.git.

## Notes

Supplementary electrochemical measurements (EIS and rate performance); feature group definitions and computation benchmarks; dimensionality reduction; cross-validation; LOFO scheme details, regression coefficients, and model performance across rates and formulations; supplementary references (PDF)
