# Supplementary material for: Forecasting Battery Electrode Performance via Electrochemical Fluorescence Microscopy and Machine-Learning
Source: ACS Appl Mater Interfaces. 2025 Dec 3;17(50):67906–13. doi: 10.1021/acsami.5c17708 (PMC12723639; doi:10.1021/acsami.5c17708)
Supplement: Supplementary file 1 [file am5c17708_si_001.pdf]

Supporting Information: Forecasting Battery Electrode Performance via Electrochemical Fluorescence Microscopy and Machine-Learning

Karla Negrete,† Marco-Tulio Fonseca Rodrigues,‡ Daniel P. Abraham,‡ and Maureen H. Tang*,¶

†*Department of Mechanical Engineering & Mechanics, Drexel University, Philadelphia, PA*

‡*Chemical Sciences & Engineering Division, Argonne National Laboratory, Lemont, IL*

¶*Department of Chemical & Biological Engineering, Drexel University, Philadelphia, PA*

E-mail: mhtang@drexel.edu

Supplementary Figures

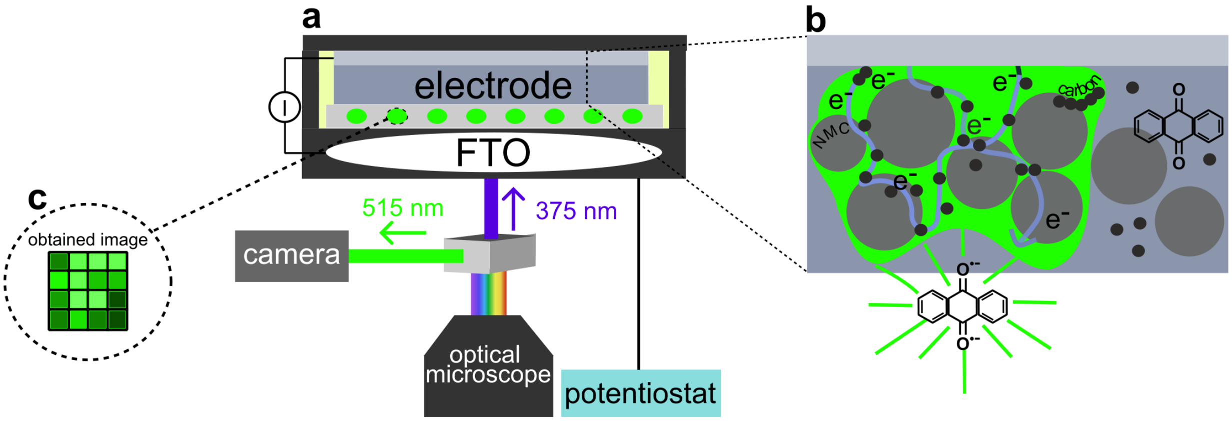

Figure S1: **Method schematic.** **(a)** Experimental configuration combining an optical microscope and potentiostat with a sealed optical cell. The cell features a transparent conductive window that serves as both the counter electrode and optical access. **(b)** Cross-section of an electrode illustrating the EFM mechanism. **(c)** Regions of interest (ROIs) undergo a 20 s reductive pulse followed by fluorescence imaging.

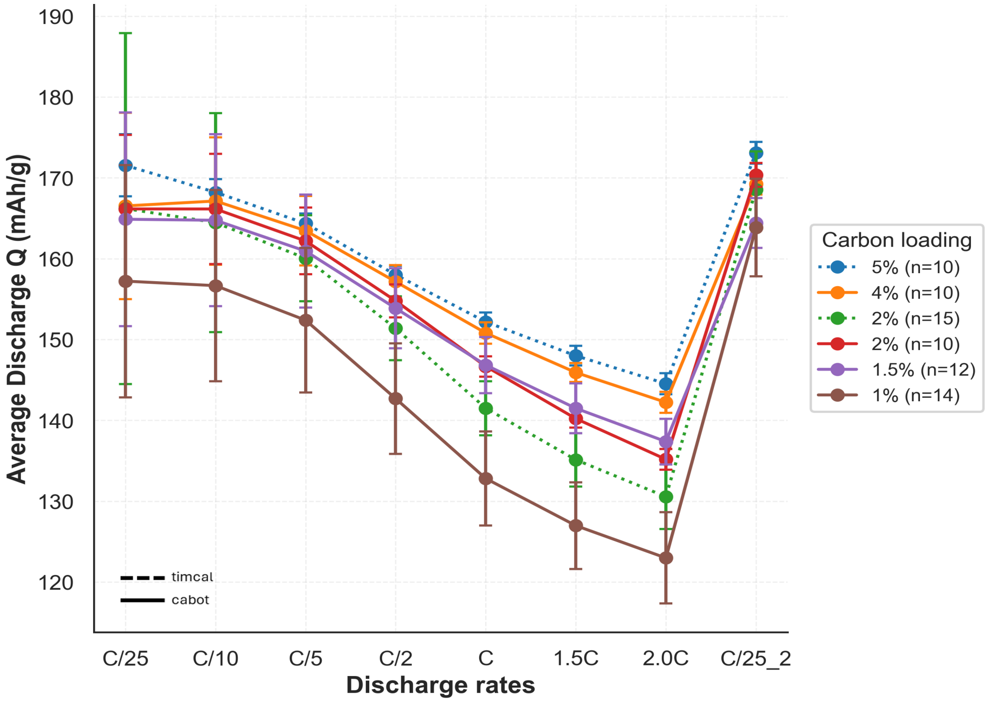

Figure S2: **Rate performance data for six NMC532 electrode compositions with varying carbon loadings.** As expected, discharge capacity declines with decreasing carbon content. Notably, electrodes using Cabot LITX 200 carbon (c) outperform those using Timcal Super C45 (t) at similar composition. Protocol stages correspond to the average of specific cycle windows: Formation (Cycles 1–3), C/25 (Cycles 4–5), C/10 (6–7), C/5 (8–9), C/2 (10–11), C (12–13), 1.5C (14–15), 2.0C (16–17), and a final C/25 step (18–19).

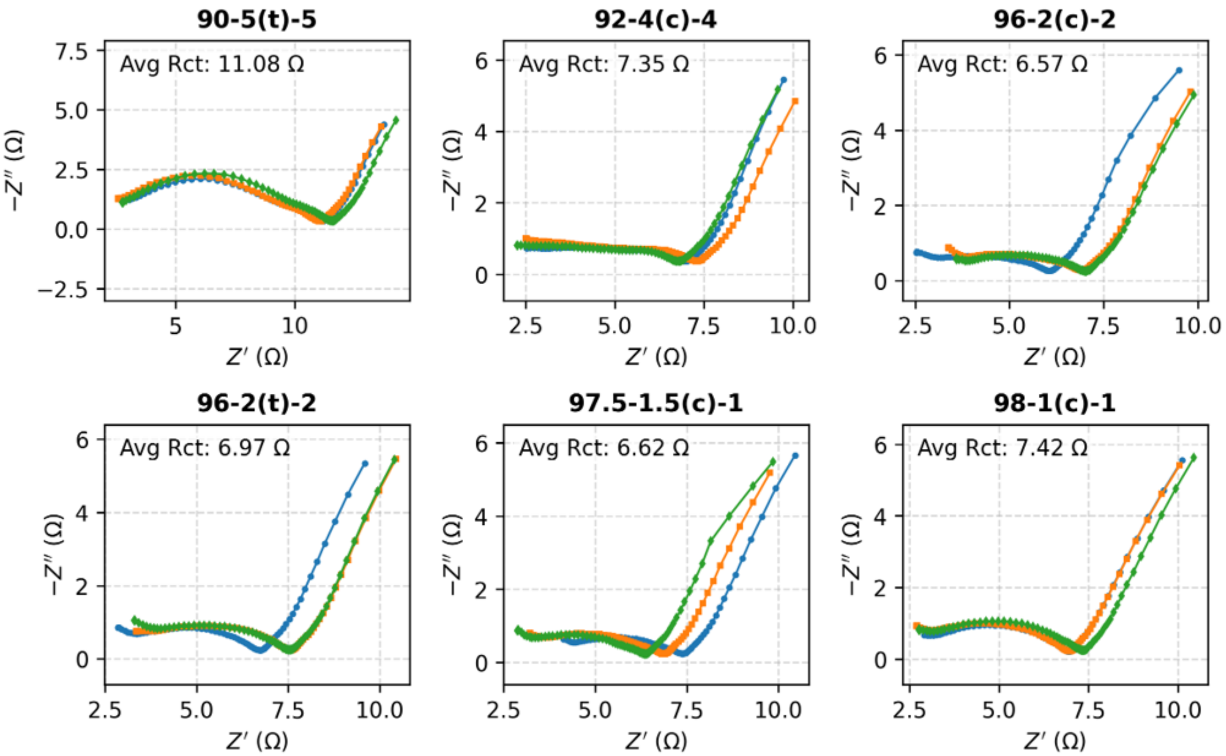

Figure S3: **Electrochemical impedance spectroscopy (EIS) data for six NMC532 electrodes.** For each composition, three electrodes were measured, and the average charge transfer resistance (R_{ct}) was determined as the width of the semicircle in the Nyquist plot ($\max(Z') - \min(Z')$) over 100 kHz to 10 mHz, excluding low-frequency contributions. Although R_{ct} includes both electrodes, differences are attributed primarily to the cathode, as the anode was consistent across cells. Notably, R_{ct} increases with carbon loading despite improved rate performance.

Table S1: Summary of image-derived heterogeneity feature groups used in the EFM–ML framework.

Feature Group	Abbrev. Prefix	# Features	Description	Source
Global Spatial Statistics	—	4	Global measures of intensity distribution, entropy, and spatial autocorrelation.	[S1,S2]
Gray Level Co-occurrence Matrix	GLCM_	17	Second-order texture descriptors from pixel co-occurrence at multiple orientations and distances.	[S3–S5]
Local Binary Patterns	LBP_	6	Encodes local pixel texture by comparing neighbors in circular patterns.	[S6,S7]
Run Length	GLRLM_	9	Measures lengths of contiguous pixel runs at different intensities.	[S8]
Gray Tone Difference	NGTDM_	5	Captures contrast and complexity between pixels and their local neighborhoods.	[S9]
Gray Level Difference Statistics	GLDS_	5	Texture measures based on gray-level differences in defined directions.	[S10,S11]
Structural Feature Matrix	SFM_	4	Descriptors derived from spatial frequency analysis of image texture.	[S12]
Line Textural Energy	LTE_	6	Line-based ternary texture descriptors over multiple orientations.	[S13]
Fourier Power Spectrum	FPS_	2	Frequency-domain summaries over radial and angular components.	[S14]
Blob Analysis	Blob	3	Morphological metrics capturing connected fluorescent domains (count, size, variability).	[S15,S16]
Patchwise Statistics	Patch_	3	Local statistical measures (mean, variance, entropy) across image patches.	[S17]

Table S2: Computation time for each image-derived heterogeneity feature group used in the EFM–ML framework.

**Feature Method**	**Avg Time (s)**	**Total Time (s)**	**Total Time (min)**	**Number of Calls**
Moran	1.362	266.93	4.45	196
Gray Level Difference Statistics (GLDS)	0.653	128.08	2.13	196
Run Length (GLRLM)	0.602	117.90	1.97	196
Fourier Power Spectrum (FPS)	0.437	85.60	1.43	196
Structural Feature Matrix (SFM)	0.375	73.41	1.22	196
Gray Tone Difference (NGTDM)	0.316	61.97	1.03	196
Line Textural Energy (LTE)	0.234	45.79	0.76	196
Blob Analysis (Blobs)	0.205	40.09	0.67	196
Local Binary Patterns (LBP)	0.157	30.73	0.51	196
Gray Level Co-occurrence Matrix (GLCM)	0.061	11.92	0.20	196
Patchwise Statistics	0.006	1.21	0.02	196
Heterogeneity Index	0.003	0.51	0.01	196
Heterogeneity Uniformity	0.001	0.21	0.00	196
Central Tendency	0.000	0.07	0.00	196

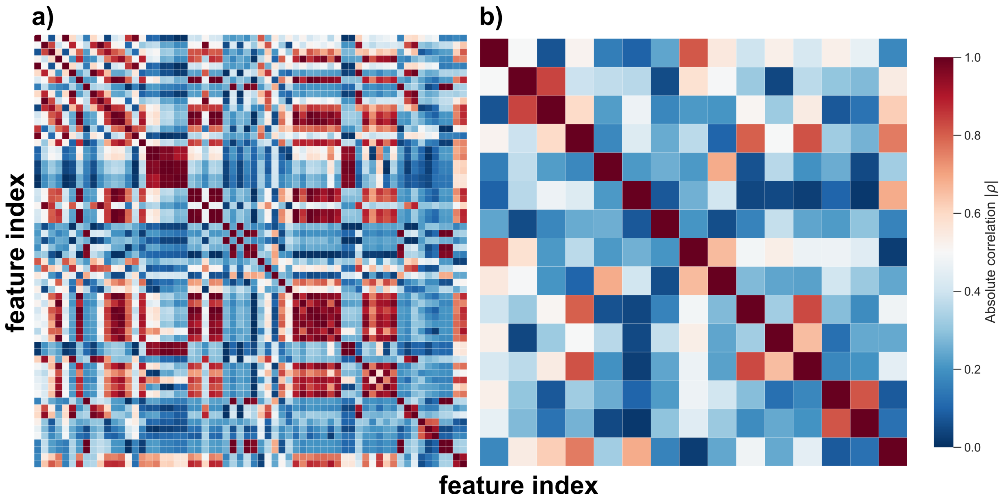

Figure S4: **Dimensionality reduction of features.** **(a)** Pearson correlation matrix of the full set of 62 extracted features. Highly correlated features ($|\rho| > 0.85$) were removed to reduce redundancy. **(b)** The resulting set of 15 uncorrelated features used for modeling.

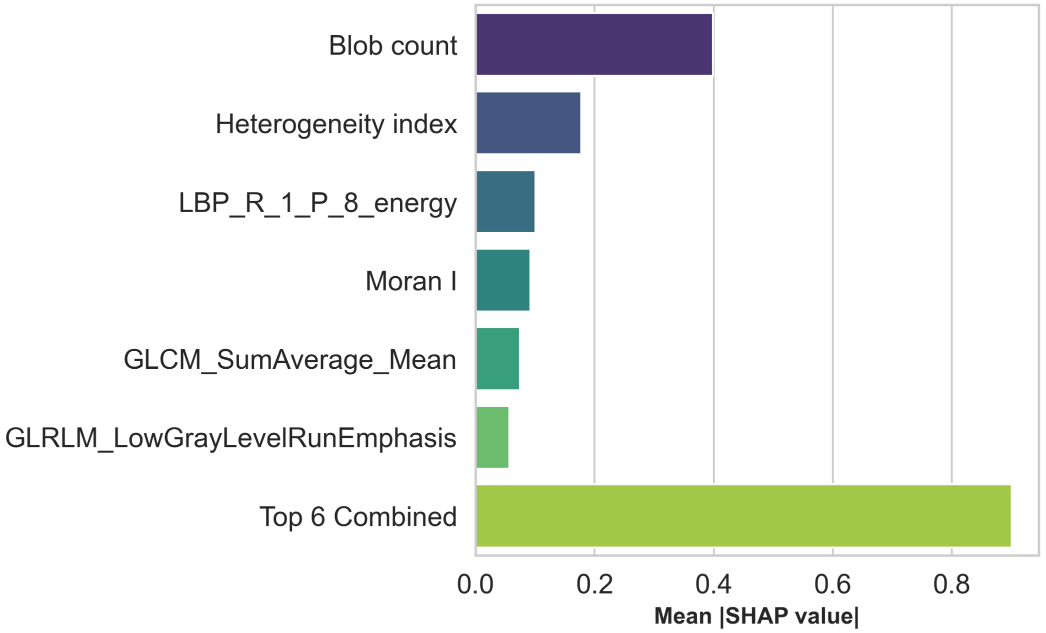

Figure S5: **SHAP analysis of key predictive features.** SHAP values quantify each feature's contribution to model output after removing correlated features ($|\rho| > 0.85$). The regularized model highlights six dominant descriptors capturing texture and morphology. Blob count exerts the strongest influence, and their combined contribution (0.9) underscores their collective importance in predicting electrode performance.

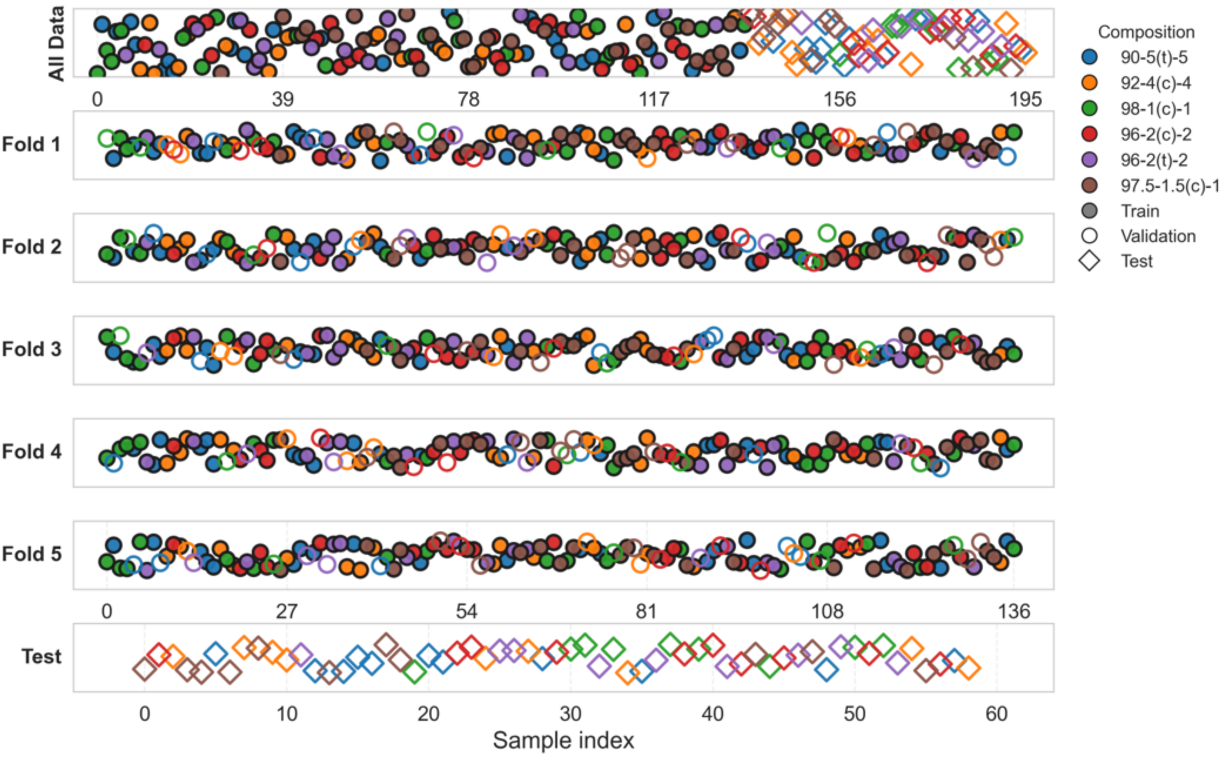

Figure S6: **Cross-validation scheme.** The data were split into 70% training and 30% test sets, with the test set held out for final evaluation (bottom panel). The training set was further partitioned using stratified 5-fold cross-validation to preserve electrode type distributions. Each fold (middle panels) designates a unique subset for validation (open circles), with the remainder used for training (filled circles). This ensures each sample is used once for validation, reducing overfitting and enabling robust hyperparameter selection. The final model was retrained on the full training set before evaluation on the independent test set.

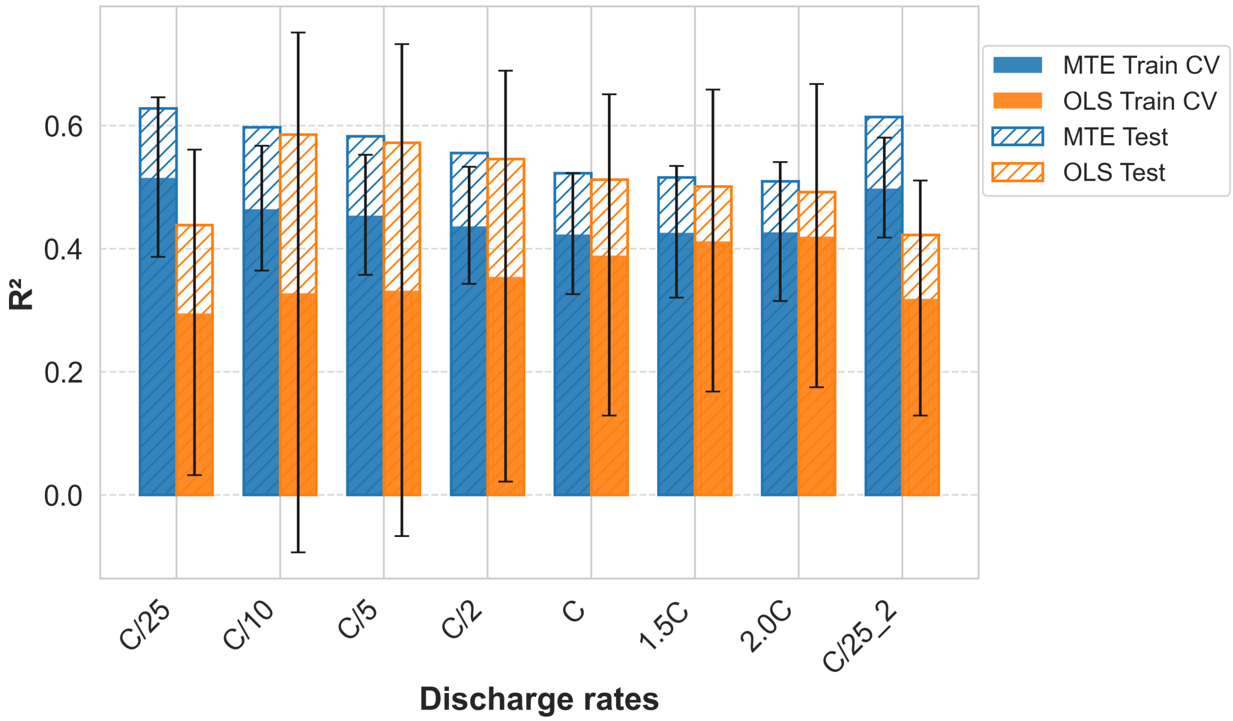

Figure S7: **Comparison of model performance across discharge rates.** R^2 values are shown for multi-task Elastic Net (MTE, blue) and ordinary least squares (OLS, orange) models. Solid bars denote mean R^2 from stratified cross-validation on the training set, with error bars indicating the standard deviation across folds. Hatched bars represent performance on the held-out test set. Across discharge rates, MTE consistently outperforms OLS, particularly at lower rates (C/25–C/5), where capacity retention is more sensitive to electrode composition. Error bars highlight variability due to limited dataset size, underscoring the importance of regularization in stabilizing model performance.

Table S3: Regression coefficients for discharge capacity (y) models by target condition from the final multitask ElasticNet model used for testing. Coefficients are rounded to three significant figures. A dash (—) indicates the feature was not included in the model for that target.

Target	Intercept	LBP_R_1_P_8_energy	Blob count	Blob area mean	GLCM Contrast Mean	Patch Variance
C/25	215	1.91	0.014	-1.77	—	—
C/10	217	1.20	0.033	-1.81	-0.136	0.0081
C/5	213	1.53	0.034	-1.87	-0.152	0.0095
C/2	213	2.84	0.047	-2.35	-0.222	0.014
C	204	5.44	0.065	-2.83	-0.323	0.022
1.5C	182	7.42	0.089	-2.79	-0.365	0.025
2.0C	161	8.72	0.111	-2.62	-0.383	0.027
C/25_2	179	2.62	0.044	-0.926	—	—

Table S4: Performance metrics of the multitask ElasticNet model for each protocol step and overall.

**Target**	**Test R^2**	**RMSE**	**Mean % Error**
C/25	0.628	2.56	1.15
C/10	0.598	2.32	1.12
C/5	0.583	2.47	1.25
C/2	0.555	3.30	1.78
C	0.523	4.38	2.55
1.5C	0.516	4.78	2.91
2.0C	0.509	4.97	3.13
C/25_2	0.614	2.06	0.94
**Overall**	**0.924**	**3.53**	**1.85**

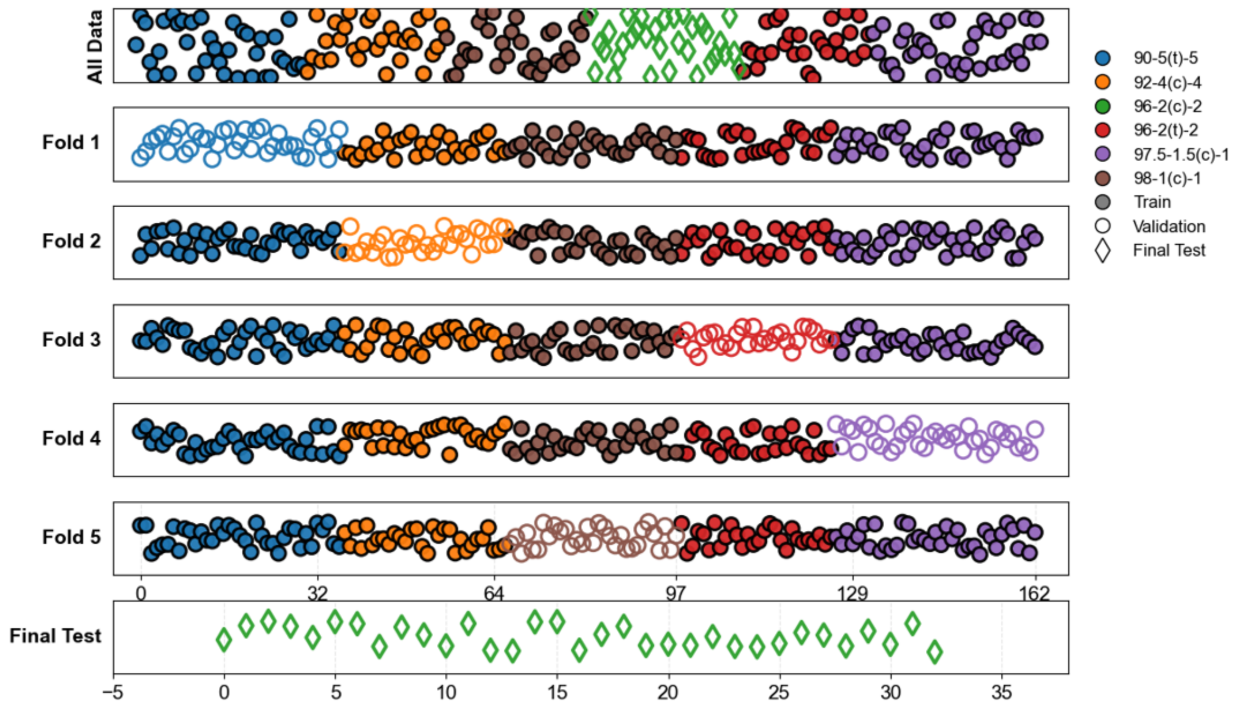

Figure S8: **Leave-one-formulation-out cross-validation scheme.** All available samples are shown at the top, with the formulations reserved for final testing (green diamonds) excluded from cross-validation. For each fold (middle panels), one formulation was held out for validation (open circles) while the remaining formulations were used for training (filled circles). This ensures that model generalization is evaluated on entirely unseen compositions rather than just shuffled subsets. After cross-validation, the model was retrained on all training and validation data before a final evaluation on the held-out formulations (bottom panel).The composition 96-2(c)-2 was chosen for the final test set because it was represented by two formulations differing only in conductive carbon type, providing a stringent assessment of whether the model generalized across material variants rather than memorizing composition labels.

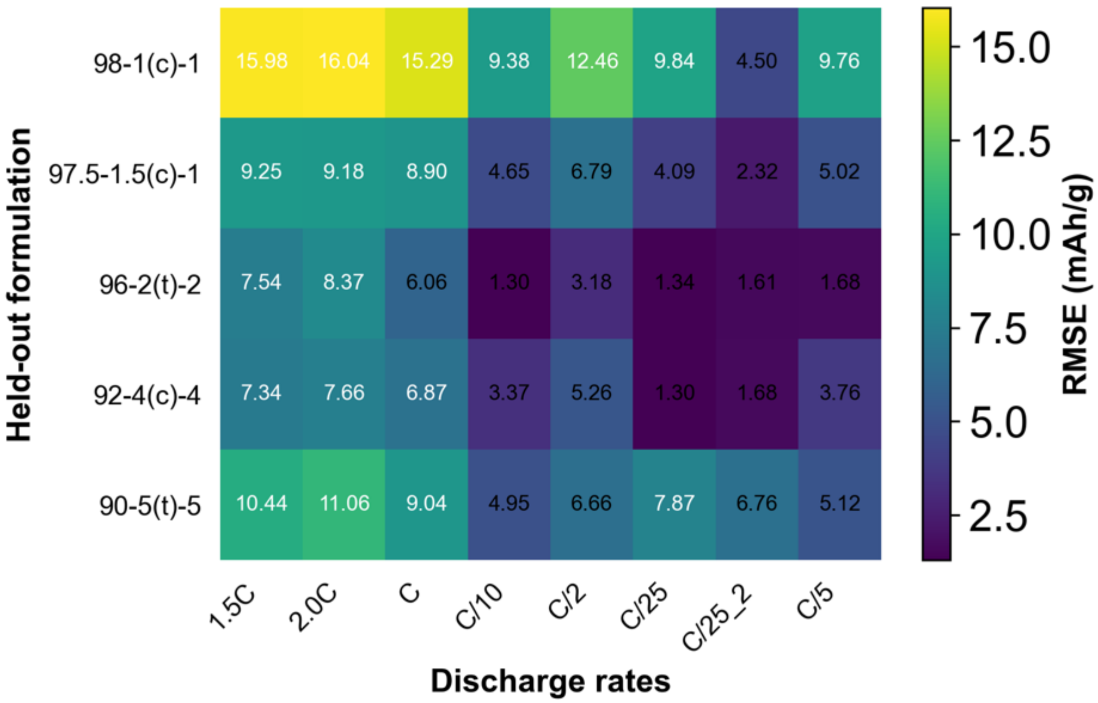

Figure S9: **Per-formulation and per-rate prediction errors under the leave-one-formulation-out scheme.** Heatmap of root mean squared error (RMSE, mAh/g) for each held-out formulation (rows) at different discharge rates (columns). Errors are lowest at intermediate rates (C/10–C/25) and highest at the extremes (1.5C–2.0C and C/5). Formulations at the limits of carbon loading (98-1(c)-1 and 90-5(t)-5) show consistently larger errors, reflecting their distinct behavior relative to the training set.

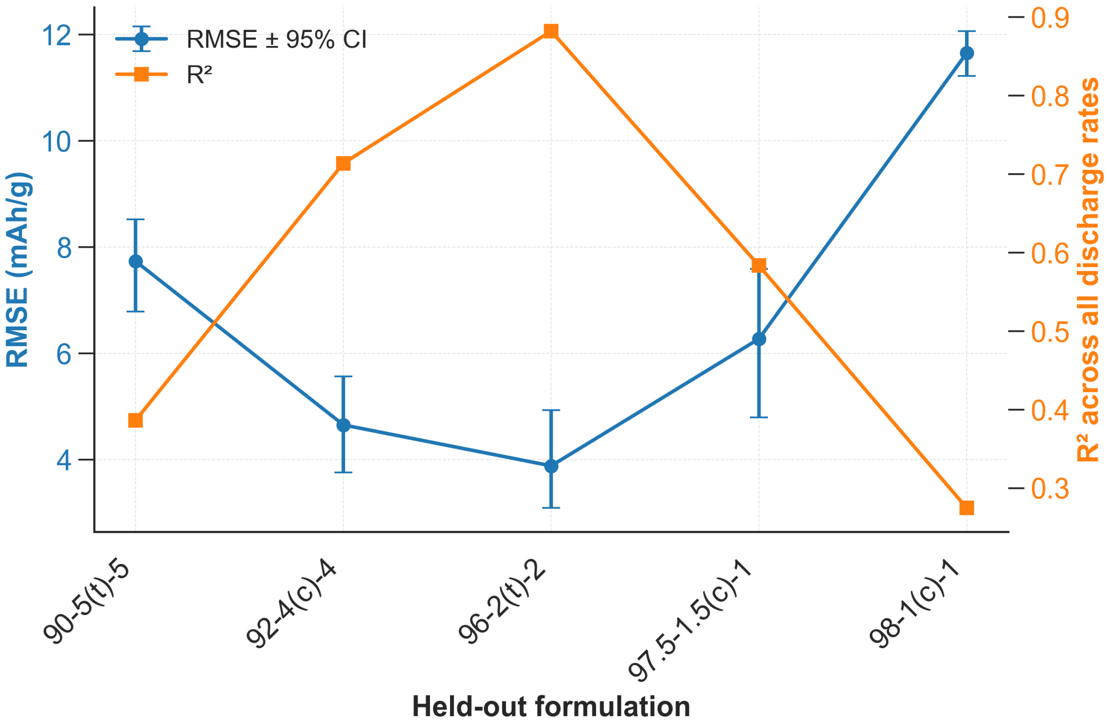

Figure S10: **Per-formulation leave-one-formulation-out results.** For each held-out formulation, the model was trained on all others and evaluated across experimental rates. Test performance is shown as the coefficient of determination (R^2) and root mean squared error (RMSE) with 95% confidence intervals. Predictions were least accurate for the formulations at the extremes of carbon loading (5% and 1%), reflecting model inability to extrapolate beyond training data.

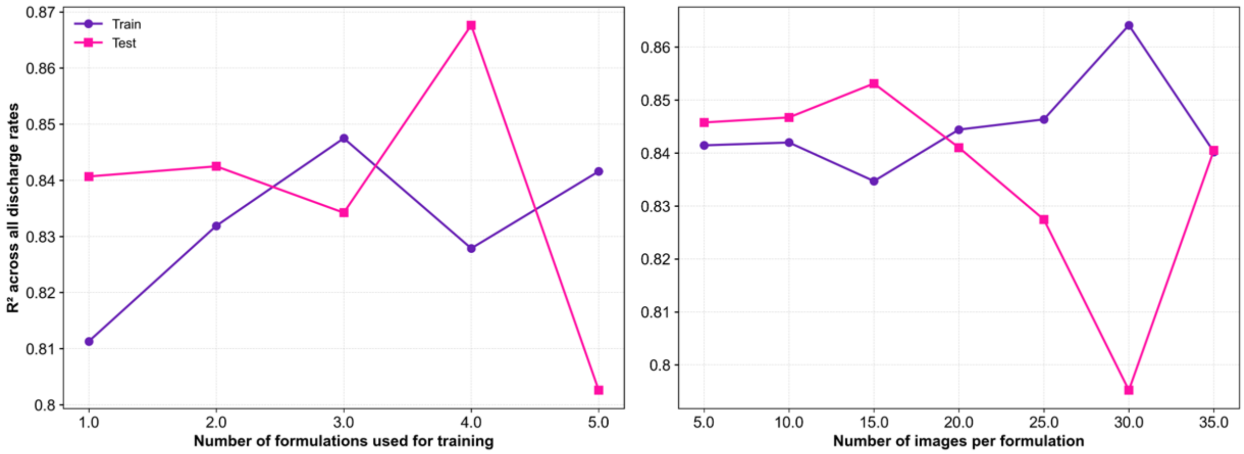

Figure S11: **Leave-one-formulation-out scheme learning curves. Left:** Training and test R^2 as a function of the number of formulations used for training. Test performance improves and approaches training performance as more formulations are included. Performance peaks when the training set contains all but two formulations, which ensures sufficient variance in the test set. Beyond this point, the test set becomes too small and the model fails to generalize. **Right:** Training and test R^2 as a function of the number of images per formulation. Both curves converge and plateau after 20 images, showing limited gains from adding further replicates.

References

(S1) Thompson, E. S.; Saveyn, P.; Declercq, M.; Meert, J.; Guida, V.; Eads, C. D.; Robles, E. S. J.; Britton, M. M. Characterisation of Heterogeneity and Spatial Autocorrelation in Phase Separating Mixtures Using Moran's I. *Chemical Engineering Science* **2017**, *173*, 42–50, Published online 1 November 2017.

(S2) Shannon, C. E. A Mathematical Theory of Communication. *Bell System Technical Journal* **1948**, *27*, 379–423.

(S3) Tang, T. T.; Zawaski, J. A.; Francis, K. N.; Qutub, A. A.; Gaber, M. W. Image-based Classification of Tumor Type and Growth Rate using Machine Learning: A Preclinical Study. *Scientific Reports* **2019**, *9*, 12529.

(S4) Mostaço-Guidolin, L. C.; Ko, A. T.; Wang, F.; Xiang, B.; Hewko, M.; Tian, G.; Ma-

jor, P. W.; Sowa, M. G. Collagen Morphology and Texture Analysis: From Statistics to Classification. *Scientific Reports* **2013**, *3*, 2190.

(S5) Haralick, R. M.; Shanmugam, K.; Dinstein, I. Textural Features for Image Classification. *IEEE Transactions on Systems, Man, and Cybernetics* **1973**, *SMC-3*, 610–621.

(S6) Lan, S.; Li, J.; Hu, S.; others A neighbourhood feature-based local binary pattern for texture classification. *The Visual Computer* **2024**, *40*, 3385–3409.

(S7) Pietikäinen, M.; Hadid, A.; Zhao, G.; Ahonen, T. *Computer Vision Using Local Binary Patterns*; Computational Imaging and Vision; Springer, London, 2011; Vol. 40; pp 13–47.

(S8) Zhang, H.; Hung, C.-L.; Min, G.; Guo, Y.; Xu, Y.; Xie, X.; Chen, Z. GPU-Accelerated GLRLM Algorithm for Feature Extraction of MRI. *Scientific Reports* **2019**, *9*, 10883.

(S9) Chen, S.; Harmon, S.; Perk, T.; Gopal, A.; Greene, R. T.; Senan, S.; Lambin, P. Using Neighborhood Gray Tone Difference Matrix Texture Features on Dual Time Point PET/CT Images to Differentiate Malignant from Benign FDG-Avid Solitary Pulmonary Nodules. *Cancer Imaging* **2019**, *19*, 56.

(S10) Gómez, W.; Pereira, W. C. A.; Infantosi, A. F. C. Analysis of co-occurrence texture statistics as a function of gray-level quantization for classifying breast ultrasound. *IEEE transactions on medical imaging* **2012**, *31*, 1889–1899.

(S11) Costa, W. S.; Haralick, R. M. Predicting expected gray level statistics of opened signals. CVPR. 1992; pp 554–559.

(S12) Wu, C.-M.; Chen, Y.-C. Statistical feature matrix for texture analysis. *CVGIP: Graphical Models and Image Processing* **1992**, *54*, 407–419.

(S13) Laws, K. I. Texture energy measures. Proc. Image understanding workshop. 1979; pp 47–51.

(S14) Matsuyama, T.; Miura, S.-I.; Nagao, M. Structural analysis of natural textures by Fourier transformation. *Computer vision, graphics, and image processing* **1983**, *24*, 347–362.

(S15) Razlighi, Q. R.; Stern, Y. Blob-like feature extraction and matching for brain MR images. 2011 Annual International Conference of the IEEE Engineering in Medicine and Biology Society. 2011; pp 7799–7802.

(S16) Moeslund, T. B. *Introduction to Video and Image Processing: Building Real Systems and Applications*; Springer, 2012; pp 103–115.

(S17) Song, Y.; Cai, W.; Zhou, Y.; Feng, D. D. Feature-based image patch approximation for lung tissue classification. *IEEE transactions on medical imaging* **2013**, *32*, 797–808.
